# The influence of aerobic fitness on obesity and its parent-offspring correlations in a cross-sectional study among German families

**DOI:** 10.1186/s12889-015-2013-x

**Published:** 2015-07-11

**Authors:** Ronja Foraita, Mirko Brandes, Frauke Günther, Karin Bammann, Iris Pigeot, Wolfgang Ahrens

**Affiliations:** Department of Biometry and Data Management, Leibniz Institute for Prevention Research and Epidemiology – BIPS, Achterstraße 30, 28359 Bremen, Germany; Institute of Sport Sciences, Carl von Ossietzky University of Oldenburg, Postfach 2503, 26111 Oldenburg, Germany; Institute of Public Health and Nursing Science (IPP), University of Bremen, Grazer Straße 4, 28359 Bremen, Germany; Faculty 3, University of Bremen, Bibliothekstraße 1, 28359 Bremen, Germany; Department of Epidemiological Methods and Etiological Research, Leibniz Institute for Prevention Research and Epidemiology – BIPS, Achterstraße 30, 28359 Bremen, Germany

**Keywords:** Cardio-respiratory fitness, Familial aggregation, Heritability, IDEFICS study, Physical activity

## Abstract

**Background:**

Overweight/obesity is an important public health burden worldwide, increasing the risk for the development of cardiovascular diseases or the metabolic syndrome. This risk may be reduced by a good aerobic fitness (AF) that can be improved by physical activity but is also influenced by genetic factors. The aim of this study was to test for familial aggregation of AF measured by maximal oxygen uptake (VO_2max_) and to estimate its heritability. Furthermore, an exploratory analysis of the association between overweight/obesity and AF was performed. In contrast to previous studies, all analyses were adjusted for additional environmental and behavioral factors, in particular for objectively measured physical activity (PA) in addition to body mass index (BMI).

**Methods:**

79 families (157 parents, 132 children) performed a maximum exercise test (spiroergometry) to assess maximum oxygen uptake. PA was measured by accelerometry. Familial aggregation of AF was determined using a two-step design: AF was adjusted for age, sex and age*sex using linear regression. Afterwards, the residuals were used to determine the intraclass correlation coefficient (ICC) by ANOVA. Heritability and associations were estimated by generalized linear mixed models.

**Results:**

Familial aggregation of AF (ICC = 0.22, *p* < 0.001) was significant but decreased when adjusted for PA or BMI. Its heritability was estimated as 40 % (adjusted for PA) using the mid-parent-offspring design. Relative to the middle quintile of AF residuals, the odds of being overweight/obese were three- to tenfold reduced in the upper quintile (adjusted for age, sex, age*sex, PA).

**Conclusions:**

AF clustered in families after controlling for PA, BMI and parental smoking. Heritability was stronger for mother-child pairs as compared to father-child pairs after controlling for PA and BMI. Above average AF was negatively associated with overweight/obesity.

## Background

Over the last few decades, the prevalence of overweight and obesity has increased worldwide [1] and remained at a high level in some countries [2, 3]. Besides physical activity (PA), improvement of aerobic fitness (AF) may become an important target for the prevention of overweight and obesity and related disorders like cardiovascular diseases, type 2 diabetes or the metabolic syndrome [4–6]. Obese people with low levels of cardiopulmonary fitness showed a higher mortality rate than obese people exhibiting higher levels of cardiopulmonary fitness [7, 8]. Being fit may thus reduce the sequelae of obesity [7, 9].

PA is defined as any muscular activity that leads to an increase of energy expenditure above resting energy expenditure, whereas AF is an estimate of the capacity of the cardiovascular systems, such as delivering oxygen to the skeletal muscles for re-synthesizing adenosine triphosphate, an essential requirement for any muscular activity [10, 11]. AF is commonly measured by maximum oxygen uptake (VO_2max_) as a measure of maximal aerobic capacity. It can be improved through regular exercise but there is high variation in the responsiveness to standardized training programs [12]. This considerable variation in the trainability of individuals and the observation that VO_2max_ tends to aggregate in families [13, 14] led to the assumption that AF has a heritable component.

The role of genetic, lifestyle, behavioral and/or environmental factors on VO_2max_ has been investigated using various approaches. Whereas early twin studies from Klissouras et al. estimated heritability to be as high as 90 % [15, 16], others suggested lower heritability proportions of 40–60 % [17–19]. In general, family studies suggest that less than half of the variation in VO_2max_ is genetically determined [13, 20–22]. The observed differences may result from statistical variation, different study designs, populations and/or statistical models. Only a few studies investigated familial aggregation of AF while controlling for confounding by weight status [20], PA [13, 14] or smoking behavior [13] although these factors are probably associated with AF [10, 23, 24]. In addition, PA was not objectively measured in these studies. Thus, unadjusted estimates of familial aggregation of AF may be inflated due to common environmental or behavioral factors.

This manuscript focusses on three research questions. The main one is whether AF aggregates in families and whether this familial aggregation is influenced by body mass index (BMI) and behavioral factors like PA. Thus, we firstly investigated the familial aggregation of AF using two-parent families with at least one biological child in the age of 6 to 17 years whilst controlling for BMI and PA. Secondly, we explored the heritability of AF whilst adjusting for BMI and PA and we thirdly assessed the association between AF and overweight/obesity whilst adjusting for PA and smoking.

## Methods

### Study sample

The study was designed as a cross-sectional study, enrolling the families of index children whose AF had been measured within the framework of the German part of the IDEFICS (Identification and prevention of Dietary- and lifestyle-induced health Effects In Children and infantS) study [25, 26]. To be eligible, these index children had to be older than five years and had to have performed a shuttle-run test in the IDEFICS study showing either high (above 70th percentile) or low (below 30th percentile) AF. Participants were selected from the extremes of the AF distribution to sharpen the contrast. Only first degree biological relatives including father, mother and siblings aged 5–17 years were eligible for this study. Participation of both parents and of at least one child, i.e. of a complete nuclear family, was required for inclusion of the respective family in the analysis. Subjects were not eligible if: (i) he/she suffered from serious heart disease or severe asthma, (ii) he/she was not capable of taking part in a maximum stage exercise test due to physical handicap, body height above 210 cm or body weight above 150 kg.

Overall, 278 families were eligible for participation in this study; 199 families did not participate for the following reasons: no time (*N* = 57), unavailability of one parent during the recruitment period (*N* = 36), not convinced of the study purpose (*N* = 3), other reasons (*N* = 56) or because they were never reached personally (*N* = 47). Eventually, 79 families with 158 adults and 137 children participated in the study (participation proportion 28 %). Six participants did not successfully perform the maximum stage test to assess their AF and were subsequently excluded: one father because of an unexpected high resting pulse rate, one child because he/she did not understand the task, two children because they belatedly refused to take part and further two children because they did not meet the workout criteria described below. Overall, a total of 289 subjects were enrolled in the study. The average family size was 3.66 with 38 families participating with one child, 34 families participating with two children and seven families participating with more than two children. This final sample provided 80 % power to detect a minimum intraclass correlation coefficient for AF of 0.14.

All examinations took place under supervision of a physician between March and August 2009. The study was approved by the ethics committee of the University of Bremen and written informed consent was given by all parents for themselves and for their children. In addition, oral assent was obtained from all participants.

### Anthropometry

Measurement of body weight and assessment of body fat percentage based on bioelectrical impedance was performed using an adapted version of electronic scale TANITA BC 420 SMA. Measurement was done non-fasted with bare feet and in underwear. Height was measured using a telescopic stadiometer, waist and hip circumferences were measured with a tape SECA 200, both following the International Standard for Anthropometric Assessment [27]. The calculated body mass index (BMI = weight[kg]/height^2^[m^2^]) was classified according to the international reference values available from the WHO [28] for participants older than 18 years. The IOTF reference [29] was used to classify childhood BMI categories. Waist-to-hip ratio was calculated dividing waist circumference [cm] by hip circumference [cm].

### Aerobic fitness

AF expressed as the maximum oxygen consumption (VO_2max_) was assessed by a maximum exercise test performed on a stationary bicycle (Ergoselect 200p). Prior to the test, each subject underwent a medical examination and an interview by a physician including a medical history with special focus on cardiovascular diseases and current medications. The measurement of AF followed the recommendations of the American Heart Association [30]. Different protocols were applied for the exercise test depending on age and sex and for children additionally depending on their weight: Fathers started with an initial workload of 50 Watts (W) which was increased by 50 W every three minutes, whereas mothers started with an initial workload of 40 W which was increased by 40 W every three minutes. All children started with an initial workload of 20 W, which was increased by 0.5 W per kg body weight every two minutes. All subjects performed the test until voluntary exhaustion. During the test, expired gas was analyzed for concentrations of O_2_ and CO_2_ (MetaMax 3B, Cortex Biophysik GmbH, Germany). Breath-by-breath values were processed by a three-point median filter to exclude outliers and were averaged over 15 s intervals. Finally, VO_2max_ was related to body weight and expressed as ml ∙ min^-1^ ∙ kg^-1^, a well-established approach to normalize VO_2max_. The test result was considered acceptable if the parents met one of the following two criteria: maximum heart rate (HR) in beats per minute of at least 200 – age in years or a respiratory exchange ratio (RER) of at least 1.0. In children, maximum voluntary exhaustion was assumed when a maximum HR of at least 185 beats per minute was reached. HR and RER were continuously displayed and recorded during the test. A 12-channel ECG was recorded during the test to control for pathological responses of the heart during exercise. Maximum workload in W was assessed by linear approximation if the last stage of the test was not maintained for the full allocated time. For example, if a father aborted the test after 1:30 min while cycling with a loading of 250 W, maximum workload was set to 200 W + (50 W ∙ 0.5) = 225 W.

### Physical activity

PA was measured using an uniaxial accelerometer (Actigraph or Actitrainer) which is described in detail in Sirard et al. [31]. The participants were asked to wear the accelerometer on the right hip for seven consecutive days during waking hours. The activity monitor recorded the number of activity counts in 15 s epochs. Consecutive zero counts of over 20 min were classified as non-wearing time. A day was considered as valid if the total minutes of wearing time (minutes the accelerometer monitored any counts) was greater than ten hours. A minimum of two valid days was required for PA variables to be included in the analysis. Average minutes per day spent in moderate or vigorous physical activity (MVPA) were calculated as average time per day spent above ≥3000 counts per minute. With this cut-off value, we chose a compromise between the many different recommended cut-points for children, adolescents and adults. Average wearing time is used to adjust for the proportion of MVPA since the time spent in MVPA is correlated with accelerometer wearing time.

### Smoking behavior

Parents were also asked to report their smoking behavior. Non-smokers were defined as parents that had never smoked and ex-smokers were defined as parents that had stopped smoking more than a year ago. Pack-years were calculated as the product of smoking duration in years and dose in packs per day (20 cigarettes ∙ package^-1^). A family was considered to be a smoking household if at least one parent stated that someone smoked at home.

### Statistical analysis

Referring to the first research question, familial aggregation of AF was assessed in complete nuclear families using the intraclass correlation coefficient (ICC) obtained from an analysis of variance (ANOVA) [32]. A significant *F*-value implies that members of a family are more alike in their AF than non-members are. The ranges of raw VO_2max_ values differ between children and adults as well as between males and females. However, comparability of AF values in children and adults as well as between sexes is required to allow analyses of familial aggregation and heritability. To make the raw AF values comparable across all study participants, a two-step design was used where in the first step a linear regression was fitted with relative VO_2max_ as the dependent variable. The resulting residuals, that are the differences between estimated and observed values of the relative VO_2max_, were included as the dependent variable in an ANOVA to determine the ICC for AF in the second step [13, 22]. More detailed, the basic linear regression model for relative VO_2max_ (step 1) included sex (*X*_1_), children’s age (*X*_2_, continuous, zero for parents), parents’ age (*X*_3_, continuous, zero for children), and two multiplicative interaction terms for age and sex as independent variables

$$ rel\kern0.5em V{O}_{2 \max }=\alpha +{\beta}_1{X}_1+{\beta}_2{X}_2+{\beta}_3{X}_3+{\beta}_4{X}_1{X}_2+{\beta}_5{X}_1{X}_3+\varepsilon $$

where the error term *ε* was assumed to be normally distributed with zero mean. Children’s age and parents’ age were modelled as two distinct continuous variables because age would appear as a bimodal variable otherwise. Other covariates like PA (average MVPA, valid time, both continuous), BMI (categorical), smoking household (binary) and a multiplicative interaction term for BMI and average MVPA (continuous) were added to the basic model as additional independent variables resulting in six different models (Table 2). Residuals from each of these models were used to calculate the ICC by an ANOVA in a second step, where each family formed one cluster. Significance tests of the ICC were adjusted for multiple testing using Bonferroni correction for six models, i.e. the significance levels was chosen as α = 0.05/6 = 0.00833. The sample size for the ICC analysis was reduced by two participants due to the restriction to complete nuclear families. Because a complete case analysis was performed, sample sizes varied due to missing values for PA items and smoking information.

To explore the heritability of AF in complete nuclear families (second research question), the mid-parent-offspring design *a*s well as the single-parent-child design were used [33]. Heritability was estimated by regressions of offspring phenotype on, both, the average phenotype of both parents (mid-parent-offspring design) and the phenotype of only one parent (single-parent-child design). The single-parent-child design allows distinguishing maternal from paternal effects. A linear mixed model of offspring AF on parental AF (*X*) was performed for the mean of the AF of both parents (mid-parent-offspring design)

$$ offspring\;AF=\alpha +\beta\;X+\gamma\;Z+\varepsilon $$

and adjusted for children’s individual PA (average MVPA, valid time) and children’s BMI where *ε* and *γ* were assumed to be normally distributed with zero mean. Families (*Z*) were taken into account as clusters and were treated as a random variable in mixed models. AF was included in the linear mixed model as the obtained residuals from the basic model on the relative VO_2max_ (see step 1 above). The estimated regression coefficient *β* represents the upper-limit estimate of the heritability (h^2^) for the mean parental AF and reflects the resemblance between first-degree relatives. Using a single-parent design, the upper-limit estimate is equivalent to twice the regression coefficient [33].

To answer the third research question, logistic mixed models were calculated to estimate the association between AF (quintiles, *W*) and overweight/obesity (*Y*). Families or respective parents were taken into account as clusters (*Z*)

$$ \mathrm{logit}\left(P\left(Y=1\Big|W,\gamma \right)\right)=\alpha +\beta \kern0.28em W+\gamma \kern0.28em Z $$

where *γ* was assumed to be normally distributed with zero mean. Residuals were classified into quintiles mainly for two reasons: 1) there are no meaningful cut-offs to classify residuals; 2) we observed a non-linearity of the odds ratios that would not have become visible if we had modeled AF using a continuous covariate. As complete nuclear families were not essential for this analysis, all 289 participants were used to estimate individual odds ratios (OR) and their 95 % confidence intervals (CI) to measure the strength and direction of the association. We performed a sub-group analysis for parents to additionally adjust for smoking behaviors.

## Results

Basic demographic and anthropometric characteristics for the 289 participants are shown in Table 1. Children’s age ranged from 6 to 17 years (mean = 9.89 years, SD = 2.29, for boys and mean = 10.02 years, SD = 2.14, for girls). Parents’ age ranged from 30 to 54 years for fathers (mean = 43.02 years, SD = 5.16) and from 31 to 48 years for mothers (mean = 40.74 years, SD = 4.12). While girls were more likely to be overweight/obese than boys (24 % for girls vs. 7 % for boys), women were less likely to be overweight/obese (49 %) than men (71 %). The minimum accelerometer wearing time (at least 10 h in 2 days) was achieved by 255 subjects, of whom 90 % (227) had a wearing time of at least 5 days and 50 % (127) of at least 7 days.

**Table 1:** Demographic and anthropometric characteristics of 289 family members included in the analysis

	Fathers (*N* = 78)	Mothers (*N* = 79)	Sons (*N* = 70)	Daughters (*N* = 62)
Age group (N (%))				
6 - <9 years			27 (38.6)	22 (35.5)
9 - <12 years			33 (47.1)	29 (46.8)
12 - <15 years			4 (5.7)	9 (14.5)
15 - <18 years			6 (8.6)	2 (3.2)
30 - <40 years	20 (25.6)	31 (39.2)		
40 - <50 years	52 (66.7)	48 (60.8)		
50+ years	6 (7.7)	0		
Age in years (mean (SD))	43.02 (5.16)	40.74 (4.12)	9.89 (2.29)	10.02 (2.14)
Height in cm (mean (SD))	179.01 (7.60)	167.05 (6.57)	141.44 (13.57)	142.71 (13.09)
Weight in kg (mean (SD))	87.31 (14.63)	73.04 (14.08)	34.41 (10.60)	37.94 (12.43)
BMI categories (N (%))^#^				
Underweight	1 (1.3)	0	8 (11.4)	5 (8.1)
Normal	22 (28.2)	40 (50.6)	57 (81.4)	42 (67.7)
Overweight	41 (52.6)	23 (29.1)	5 (7.1)	10 (16.1)
Obese	14 (17.9)	16 (20.3)	0	5 (8.1)
Waist-to-hip ratio (mean (SD))	0.93 (0.06)	0.81 (0.06)	0.84 (0.06)	0.82 (0.06)
Arm circumference in cm (mean (SD))	33.99 (3.60)	31.28 (3.97)	21.11 (2.79)	23.01 (3.46)
Body fat percentage (mean (SD))	23.14 (5.56)	34.34 (6.54)	15.17 (4.24)	22.61 (6.64)
Aerobic fitness in relative VO_2max_	33.73 (7.02)	27.37 (4.89)	46.30 (6.78)	40.29 (5.62)
Physical activity in average minutes per day (average MVPA) (mean (SD))^*^	22.82 (13.49)	19.13 (15.21)	47.48 (25.18)	29.77 (13.48)
Smoking status (N (%))				
Non-smoker	26 (33.3)	39 (49.4)		
Smoker	31 (39.7)	19 (24.1)		
Ex-smoker^£^	20 (25.6)	20 (25.3)		
Not specified	1 (1.3)	1 (1.3)		
Pack-years (PY) (mean (SD))				
Smoker^$^	15.93 (12.11)	15.62 (11.35)		
Ex-smoker^£ ‡^	12.87 (9.08)	10.77 (6.27)		

^#^Body mass index (BMI) categories according to WHO in adults (<18.5; 18.5- < 25; 25- < 30; 30+) and IOTF in children [29]

^*^N_Fathers_ = 67; N_Mothers_ = 69; N_Sons_ = 64; N_Daughters_ = 55

^£^Parents that had stopped smoking more than a year ago

^$^N_Fathers_ = 29; N_Mothers_ = 19; N_PY not specified_ = 2

^‡^N_Fathers_ = 20; N_Mothers_ = 14; N_PY not specified_ = 6

VO_2max_ residuals adjusted for age and sex including their interactions (basic model, Table 2) led to a statistically significant ICC of 0.22 (*p* < 0.001, AF adjusted for age, sex and age*sex). The ICC decreased after an additional adjustment of AF for PA (ICC = 0.19, *p* = 0.001), for BMI (ICC = 0.14, *p* = 0.004), and for PA and BMI simultaneously (ICC = 0.15, *p* = 0.008).

**Table 2:** Familial aggregation of age, sex and age*sex adjusted aerobic fitness residuals

		ANOVA	
Regression model from which residuals were obtained for ANOVA	Number of subjects	ICC (p-Value)	R^2^
age + sex + age*sex	287	**0.22 (<0.001)**	0.43
age + sex + age*sex + PA^†^	223	**0.19 (0.001)**	0.41
age + sex + age*sex + BMI^#^	287	**0.14 (0.004)**	0.37
age + sex + age*sex + BMI^#^ + PA^†^	223	**0.15 (0.008)**	0.38
age + sex + age*sex + BMI^#^ + PA† + BMI^#^*PA^†^	223	**0.15 (0.007)**	0.38
age + sex + age*sex + BMI^#^ + PA^†^ + smoking household	219	**0.15 (0.008)**	0.38

Adjusted for multiple testing: α = 0.05/6 = 0.00833, bold numbers indicate statistically significant intraclass correlation coefficients (ICCs)

^†^Physical activity (PA) is included as two variables: average minutes per day spent in moderate or vigorous physical activity (MVPA) and average minutes of wearing time; interaction term as average minutes per day spent in MVPA*BMI

^#^Body mass index (BMI) categories according to WHO in adults (<18.5; 18.5- < 25; 25- < 30; 30+) and IOTF in children [29]

The exploratory analyses yielded the following results: The heritability of AF was estimated as 0.42 (*p* = 0.003, AF adjusted for age, sex, and age*sex) using the mid-parent-offspring design for complete nuclear families (Table 3). It varied between 0.32 (mid-parent-daughter) and 0.49 (mid-parent-son) using the mid-parent-child design and between 0.18 (mother-son) and 0.60 (father-son) using the single-parent-child design. When the mixed models were adjusted for BMI and PA, the heritability varied between 0.13 (mid-parent-daughter) and 0.43 (mid-parent-son) and between 0.08 (father-daughter) and 0.52 (mother-son) using the mid-parent-child design and the single-parent-child design, respectively.

**Table 3:** Estimated heritability of age, sex and age*sex adjusted aerobic fitness residuals

	No adjustment			Adjusted for physical activity^†^			Adjusted for BMI^#^			Adjusted for PA^†^ and BMI^#^			Adjusted for PA^†^, BMI^#^ and BMI^#^*PA^†^		
Design	N	β (SE)	h^2^	N	β (SE)	h^2^	N	β (SE)	h^2^	N	β (SE)	h^2^	N	β (SE)	h^2^
Mid-parent – offspring	131	0.42 (0.14)	0.42	118	0.40 (0.13)	0.40	131	0.31 (0.13)	0.31	118	0.33 (0.13)	0.33	118	0.33 (0.13)	0.33
Mid-parent – son	70	0.49 (0.21)	0.49	64	0.45 (0.19)	0.45	70	0.44 (0.21)	0.44	64	0.43 (0.21)	0.43	64	0.42 (0.21)	0.42
Mid-parent – daughter	61	0.32 (0.16)	0.32	54	0.28 (0.16)	0.28	61	0.14 (0.15)	0.14	54	0.13 (0.16)	0.13	54	0.13 (0.15)	0.13
Father – son	70	0.30 (0.12)	0.60	64	0.22 (0.12)	0.44	70	0.26 (0.13)	0.52	64	0.20 (0.12)	0.40	64	0.20 (0.13)	0.40
Father – daughter	61	0.22 (0.11)	0.44	54	0.13 (0.11)	0.26	61	0.08 (0.11)	0.16	54	0.04 (0.11)	0.08	54	0.03 (0.11)	0.06
Mother – son	70	0.09 (0.21)	0.18	64	0.24 (0.19)	0.48	70	0.12 (0.20)	0.24	64	0.26 (0.19)	0.52	64	0.23 (0.20)	0.46
Mother – daughter	61	0.14 (0.15)	0.28	54	0.21 (0.14)	0.42	61	0.08 (0.13)	0.16	54	0.13 (0.14)	0.26	54	0.15 (0.13)	0.30

^†^Physical activity is included as two variables: average minutes per day spent in moderate or vigorous physical activity (MVPA) and average minutes of wearing time

^#^Body mass index (BMI) categories according to WHO in adults (<18.5; 18.5- < 25; 25- < 30; 30+) and IOTF in children [29]

Higher AF was negatively associated with overweight/obesity (Table 4). Adjusted for age, sex, and age*sex, the top 20 % of the highest age, sex and age*sex adjusted residuals for relative VO_2max_ had about tenfold lower odds to be overweight/obese as compared with the middle 20 % (OR = 0.10, CI = (0.03; 0.34)). Subjects in the fourth quintile had about threefold lower odds to be overweight/obese (OR = 0.32, CI = (0.11; 0.91)). Subjects in the first and second quintile each had about twofold higher odds to be overweight/obese than the middle 20 %, although this is not a striking result from a statistical point of view. These associations persisted after adjustment for PA. Restricting the analysis to parents, subjects in the first quintile had reduced odds to be overweight/obese while the results for the other quintiles were similar to those obtained for all participants.

**Table 4:** ORs and 95 % CIs for overweight/obesity by quintiles of age, sex and age*sex adjusted aerobic fitness residuals

Adjustment in logistic mixed models		Fitness level (quintiles of VO_2max_ residuals)				
All participants	N	0-20 %	20-40 %	40-60 %	60-80 %	80-100 %
No further adjustment	289	2.01 (0.95; 4.24)	2.26 (1.06; 4.81)	1.00	0.40 (0.18; 0.91)	0.23 (0.09; 0.58)
age + sex + age*sex	289	1.94 (0.70; 5.40)	2.34 (0.86; 6.35)	1.00	0.32 (0.11; 0.91)	0.10 (0.03; 0.34)
age + sex + age*sex + PA^†^	255	1.17 (0.38; 3.62)	1.97 (0.66; 5.90)	1.00	0.27 (0.09; 0.84)	0.09 (0.02; 0.37)
Parents only						
No further adjustment	157	1.23 (0.39; 3.81)	2.05 (0.62; 6.75)	1.00	0.29 (0.10; 0.86)	0.11 (0.03; 0.36)
age + sex + age*sex	157	0.73 (0.20; 2.75)	1.88 (0.51; 6.87)	1.00	0.24 (0.07; 0.84)	0.04 (0.01; 0.17)
age + sex + age*sex + PA^†^	136	0.59 (0.13; 2.63)	1.51 (0.34; 6.64)	1.00	0.17 (0.04; 0.68)	0.04 (0.01; 0.18)
age + sex + age*sex + PA^†^ + smoking^§^	126	0.39 (0.07; 2.08)	2.27 (0.37; 14.14)	1.00	0.14 (0.03; 0.66)	0.03 (0.00; 0.17)

^†^Physical activity (PA) is included as two variables: average minutes per day spent in moderate or vigorous physical activity (MVPA) and average minutes of wearing time

^§^Pack-years

## Discussion

The main focus of the study was to assess the degree of familial aggregation of AF and to disentangle the contributions of genetic predisposition from those of the environment and (physical activity) behavior. A better understanding of the role of these different factors in determining AF may eventually facilitate the development of personalized interventions. Our study showed a significant familial aggregation of AF and a mid-parent-offspring heritability of AF of 40 %. Moreover, we found that individuals who are fitter than average have three- to tenfold reduced odds of being overweight/obese. Our findings are supported by previous studies that suggested that VO_2max_ aggregates in families [13, 14, 20, 34]. Some of these studies did not include PA as a potential confounder [20, 34], focused on a PA intervention strategy [35] or were interested in the change of AF in response to regular training [12]. In contrast to these studies, we adjusted VO_2max_ not only for BMI but also for PA and parents’ smoking behavior.

Our model including the covariates sex, age and their interaction terms explained 43 % of the variance of AF. The corresponding ICC suggested that 22 % of total variance lies between families. The ICC decreased slightly after adjustment for PA. The decrease was more pronounced after additional adjustment for BMI. This means that after adjustment for PA and/or BMI the variation of AF within families increased as compared to the variation between families. Differences between ICCs after controlling for BMI and PA are apparently caused by genetic and/or non-shared environmental or behavioral factors that have no significant effect on the covariance between family members. Similar results for familial aggregation of VO_2max_ adjusted for age, BMI and PA have been reported for Anglo- and Mexico-American families [14]. However, Sallis et al. used Pearson correlations that – in contrast to the ICC – do not account for the family structure in the data. Further adjustment for smoking household had no effect on the familial aggregation of the relative VO_2max_. We also performed ICC analyses between parents only to explore whether the familial correlation of AF is rather a consequence of common environmental than of genetic influences. A low correlation between parents despite a high familial correlation would indicate that family resemblance is more likely due to genetic factors than to a shared environment or behavior [20]. In our study, the spouse ICCs for AF ranged from -0.02 to 0.1 and none was significant (results not shown; same models as in Table 2). This result suggests that the resemblance of aerobic fitness cannot be explained by shared environmental or behavioral factors, which in turn indicates that the correlation of AF within families is due to genetic factors and/or the non-shared environment/behavior. Other studies, like the HERITAGE Family Study [20], reported spouse correlations, which might suggest a larger impact of the shared environment/behavior. However, the analysis based on the HERITAGE study only considered participants with a sedentary lifestyle at baseline. Thus, spouses with great differences in physical activity levels were not eligible and a high degree of spousal correlation was thus inherent to the study design. Additional ICC analyses indicated a significant resemblance of AF between siblings (ICC values ranging from 0.16 to 0.39). Here, the lowest value was estimated after adjustment of VO_2max_ for age, sex, BMI and PA. These results suggest that shared PA behavior and similar BMI explain most of the covariance of AF.

Next, we explored the heritability of AF and calculated upper-limit heritability estimates that capture the proportion of phenotypic variation that is due to genetic variation between individuals. The mid-parent-offspring heritability of AF was 42 % which is consistent with previous studies [20]. We found the highest heritability value for the father-son relationship reaching 60 % and the lowest heritability value for the mother-son relationship reaching 18 %. Father-son similarities regarding the maximal aerobic power were reported previously [21, 34]. Guion et al. [34] explained this phenomenon by comparable body compositions and shared activity patterns of fathers and their sons. The heritability estimates differed between the mid-parent design and the single-parent designs. After adjustment for PA and BMI, the heritability estimates decreased marginally for the mid-parent-son design and decreased substantially for the other mid-parent-offspring designs. Overall, the heritability estimates were less strong for paternal effects (father designs) and more pronounced for maternal effects (mother designs) after adjustment for BMI and/or PA. This suggests that the maternal heritability of AF is stronger than the paternal heritability. Bouchard et al. [20] hypothesized that the higher mother-child heritability is potentially due to mitochondrial inheritance.

Another question addressed by the present study was whether AF is associated with overweight/obesity. Our results suggested that higher relative maximal aerobic power was negatively associated with overweight/obesity. This negative association became even stronger when the analyses were adjusted for PA. However, associations between the lower AF quintiles and overweight/obesity were generally covered by wide confidence intervals and showed no clear trend, i.e. ORs for overweight/obesity did not monotonically decrease from the first quintile (lowest AF) to the fifth quintile (highest AF). A non-linear association between AF and BMI was also found by others [36, 37] but this may be due to reverse causation. The negative associations between AF and overweight/obesity were stronger for the fourth and fifth quintile of AF when restricted to spouses only. Adjustment for smoking behavior strengthened the association between AF and overweight/obesity among parents since heavy smokers usually have lower aerobic power [10]. Whereas our main analysis combined overweight or obese persons as cases we conducted in addition two sensitivity analyses for overweight and obese cases separately. We were able to replicate the non-linear trend for the ORs in the subsample of overweight cases, whereas the ORs showed a linear trend in the subsample of obese cases. These results suggest that it is most likely to be obese in the lowest fitness quintile, whereas it is most likely to be overweight in the second lowest quintile.

This study faces some limitations: Due to the exclusion criteria, our results may not apply to families with extremely obese members. In addition, obese boys are underrepresented in our sample compared to the German children and adolescents population according to the representative KIGGS survey (see Kurth and Schaffrath Rosario [38]). However, the overall proportion of obese/overweight children in our sample is similar to the proportion of obese/overweight children in the nationally representative KIGGS survey such that the underrepresentation of obese boys should not have a major impact on our study results. Our sampling design may hamper to transfer our results to a normal population since we examined families from children taken from the upper and lower 30th percentiles in order to increase the power to detect familial aggregation. This might in turn inflate our heritability estimates. Notwithstanding, the opportunity to select families whose children were on either extreme of the AF spectrum should maximize the spread between AF levels in our study sample and should strengthen our ability to detect a relationship between different levels of AF and overweight/obesity. The selection of families was based on a shuttle run test value, which is an approximation of VO_2max_ from reference values. In our study, we measured VO_2max_ directly by spiroergometry. To investigate whether this selection changed the uni-modal distribution of VO_2max_ into a bi-modal distribution, density plots of VO_2max_ residuals were produced for children and parents, but the uni-modality of the distribution was retained (data not shown). The low participation proportion of 28 % may impair the external validity of our findings. Nevertheless, the ability of our study to identify the factors contributing to the familial aggregation of AF and to assess its relationship with PA and overweight/obesity should not suffer from selection effects.

The radiometric approach of scaling VO_2max_ by body weight does not account for body size and body composition, which might bias the estimates of AF. Many other scaling approaches are discussed in the literature that e.g. include body fat, lean body mass, lean mass of the legs or different power functions for body mass [39, 40], but as far as we know none of them can be considered as gold standard to be used in population-based studies. However, as we did not include these measurements in our study, scaling by body weight remains the reasonable approach for our study, but potentially could slightly affect the relationship between VO_2max_ within families. Since accelerometer measurements were taken during spring and summer, PA levels may be overestimated. Different cut-points have been proposed to define moderate to vigorous PA [31, 41–44], but a definition of an optimal cut-point is still missing, especially for children [45]. Therefore, we applied the same cut-points to classify moderate to vigorous activity for all participants. We think that this approach is justified since all analyses were adjusted for age and sex. Physical maturity was not assessed in children and adolescents although pubertal stage is an important determinant of AF. But because maturation is not related to parental AF – the main variable of interest for the heritability analysis – we have to assume that pubertal stage does not confound the association between parental and children’s AF. Smoking and drinking habits were not assessed in children and adolescents. According to German smoking statistics [46, 47] we would not expect more than 3 of the 21 adolescents (12-17 years of age) in our study to smoke. In addition, Vozoris and O'Donnell [48] reported no significant differences between smokers and never smokers in estimated peak oxygen uptake after adjusting for age and sex.

## Conclusion

This study showed significant familial aggregation of aerobic fitness although resemblance attenuated after controlling for age, sex, BMI and PA measured by accelerometry. Further analyses using the same adjustment sets indicated an estimated heritability of AF of 33 %. In fact, our data supported the hypothesis that heritability is stronger for mother-child pairs as compared to father-child pairs. Additionally, we could show that an above-average high AF is associated with lower BMI regardless of PA.
